# Respiratory Syncytial Virus Prevalence and Risk Factors among Healthy Term Infants, United States

**DOI:** 10.3201/eid3010.240609

**Published:** 2024-10

**Authors:** Ferdinand Cacho, Tebeb Gebretsadik, Larry J. Anderson, James D. Chappell, Christian Rosas-Salazar, Justin R. Ortiz, Tina Hartert

**Affiliations:** Vanderbilt University Medical Center, Nashville, Tennessee, USA (F. Cacho, T. Gebretsadik, J.D. Chappell, C. Rosas-Salazar, T. Hartert);; Emory University School of Medicine and Children’s Healthcare of Atlanta, Atlanta, Georgia, USA (L.J. Anderson);; University of Maryland School of Medicine, Baltimore, Maryland, USA (J.R. Ortiz)

**Keywords:** respiratory syncytial virus, respiratory infections, RSV, risk factors, infant, United States, viruses

## Abstract

In a population-based birth cohort study of respiratory syncytial virus surveillance in the United States, 897/1,680 (53.4%) children were infected during infancy; 25 (2.8%) of those were hospitalized. Among symptomatic infants, 143/324 (44.1%) had lower respiratory tract infections. These data provide benchmarks to monitor effects of maternal vaccines and extended half-life monoclonal antibodies.

Respiratory syncytial virus (RSV) is a leading cause of illness in infants (*1*). Previous epidemiologic studies of RSV infection during infancy have focused on symptomatic illness, predominantly lower respiratory tract infections (LRTI) and hospitalizations (*2*). However, population-based surveillance studies to determine prevalence of and risk factors for RSV infection among healthy infants in the United States are lacking.

We determined prevalence of RSV infection by 1 year of age in a population-based birth cohort of healthy term infants in the United States. We excluded infants from the parent study, Infant Susceptibility to Pulmonary Infections and Asthma Following RSV Exposure (INSPIRE) (*3**,**4*), if they were not enrolled during well-child visits from 1 of 11 participating regional pediatric practices. We ascertained RSV infections by active surveillance using quantitative reverse transcription PCR (qRT-PCR) testing of nasal samples collected based on symptom surveys every 2 weeks and by passive surveillance by serum RSV antibody testing of all infants at 1 year of age during 2 RSV seasons, 2012–13 and 2013–14. If an infant met specified criteria for an acute respiratory infection, we conducted an in-person respiratory illness assessment and collected a nasal wash sample, which we used for the molecular detection of RSV by qRT-PCR. We also collected blood samples from all participating infants at 1 year of age and measured RSV serum antibody titers by ELISA using published protocols (*5*). We calculated 1-year prevalence of RSV infections, upper respiratory tract infections, LRTI, and healthcare utilization. We estimated the adjusted association and relative contribution of risk factors for RSV infection in the first year of life using multivariable logistic regression. The Institutional Review Board of Vanderbilt University approved INSPIRE, and 1 parent of each child provided written informed consent. INSPIRE methods have been published, and full study methods are available (*3*,*4*) (Appendix).

Among 1,680 infants who met inclusion criteria for our study, 897 (53.4%) were infected with RSV in the first year of life and 783 (46.6%) were not (Figure 1; Appendix Figure). Active surveillance detected 36.1% of RSV infections in symptomatic infants, whereas 63.9% were ascertained by serology alone. In all study infants, 1.5% (95% CI 0.96%–2.1%; n = 25) were hospitalized for RSV and 8.5% (95% CI 7.2%–9.9%; n = 143) had RSV LRTI. Among the subset with RSV infection, 2.8% (95% CI 1.9%–4.1%; n = 25) were hospitalized for RSV and 15.9% (95% CI 13.6%–18.4%; n = 143) had RSV LRTI. Restricted to the subset with symptomatic RSV infections meeting study criteria for a respiratory illness visit (n = 324), 55.9% had upper respiratory tract infections and 44.1% LRTIs. There were no infant deaths from RSV infection. We found that more than half of infants were RSV-infected in the first year of life. The rates of symptomatic disease, LRTI, and healthcare utilization demonstrate the considerable burden of respiratory illness in otherwise healthy infants; 1.5% of the cohort, or 2.8% of those infected, experienced RSV-associated hospitalizations.

**Figure 1:**
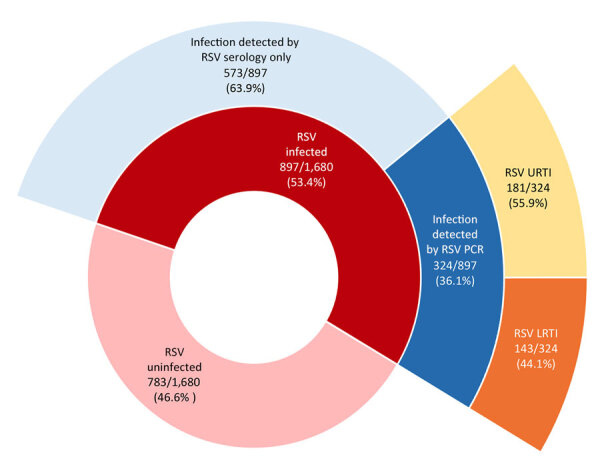
Percentages of RSV infection, symptomatic disease, LRTI, and URTI in the first year of life among healthy term infants, United States. Each ring represents the subset of the inner ring and adds to 100%. LRTI, lower respiratory tract infection; RSV, respiratory syncytial virus; URTI, upper respiratory tract infections.

Risk factors for RSV infection during infancy in order of contribution were infant birth month (June vs. referent October, OR 2.42 [95% CI 1.78–3.29]), presence of siblings (OR 1.50 [95% CI 1.22–1.84]), daycare attendance (OR 1.54 [95% CI 1.24–1.93]), increasing percentage below the poverty level in the residential neighborhood (21% vs. 8%; OR 1.19 [95% CI 1.05–1.36]), and public insurance (OR 1.28, 95% CI 1.02–1.62) (Figure 2). Secondhand smoke exposure, sex, ever being breastfed, maternal asthma, and study year were not significantly associated with likelihood of infant RSV infection. 

**Figure 2:**
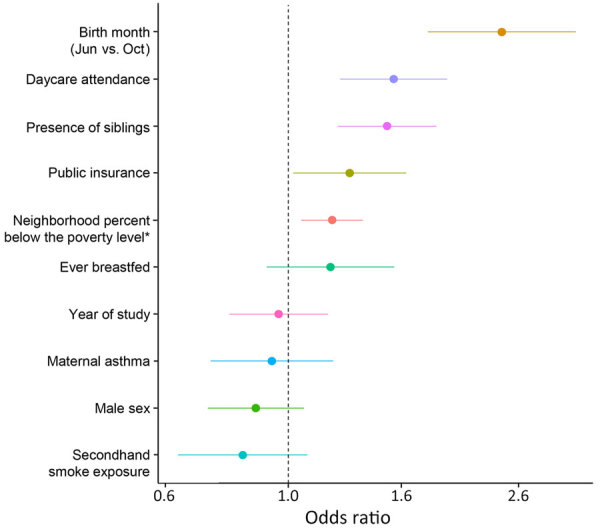
Respiratory syncytial virus infection risk factors in the first year of life in study of RSV among healthy term infants, United States. Adjusted odds ratios were estimated using multivariable logistic regression, referent is October birth month. Dots indicate odds ratio and horizontal line 95% CI. Dashed vertical line indicates the null effect. Asterisk indicates residence in a census tract with increasing percentage of people below the poverty level (interquartile range difference).

The risk factors we identified increase viral exposure and underscore the potential for nonpharmacologic interventions to prevent infection (*6*). Earlier birth month was the strongest risk factor; we speculate that parental behaviors based on infant age affect exposure intensity (e.g., age at which infants are started in daycare). The risk factors of neighborhood percentage poverty and public insurance indicate the need to address socioeconomic determinants of RSV prevention.

The first limitation of our study is that eligibility criteria and sociodemographic characteristics might not be generalizable to other populations. In addition, our cohort represents a population that may be healthier, because they were term infants; however, this group represents half of RSV hospitalizations who are eligible for RSV prevention products. There is also potential for misclassification of infants categorized as uninfected with RSV in infancy (*4*). However, the proportions of both those with symptomatic respiratory illness and overall RSV serologic positivity are very similar to estimates from other studies (*7*–*9*). Although this cohort represents 2 RSV seasons during 2012–2014, we do not expect that time period to significantly affect the rates or risk factors for infection, which were similar across both RSV seasons.

In conclusion, our data are important estimates of the burden of RSV disease and risk factors for infection in healthy term infants. Our findings provide a benchmark to monitor the effects in the United States of recently available maternal vaccines and extended half-life monoclonal antibodies for severe RSV illness prevention in early life (*10*).

Appendix Additional information about respiratory syncytial virus prevalence and risk factors among healthy term infants, United States.
