# Supplementary material for: Respiratory Syncytial Virus Prevalence and Risk Factors among Healthy Term Infants, United States
Source: Emerg Infect Dis. 2024 Oct;30(10):2199–202. doi: 10.3201/eid3010.240609 (PMC11431910; doi:10.3201/eid3010.240609)
Supplement: Appendix — Additional information about respiratory syncytial virus prevalence and risk factors among healthy term infants, United States. [file 24-0609-Techapp-s1.pdf]

Article DOI: http://doi.org/10.3201/eid3010.240609

RSV Prevalence and Risk Factors among Healthy Term Infants, United States

Appendix

Methods

Study Population

The Infant Susceptibility to Pulmonary Infections and Asthma Following RSV Exposure study (INSPIRE) is a large, population-based, birth cohort of healthy infants born at term between 2012 and 2013. INSPIRE recruited participants from 11 regional practices across middle Tennessee and the catchment zone encompassed urban, suburban, and rural areas. To be eligible for INSPIRE, infants had to be healthy overall (i.e., infants had no serious pulmonary, cardiovascular, or neurologic diseases), born at term, had a birthweight equal to or greater than 2250 g, and were born between June to December, 2012, or June to December, 2013. Thus, by study design, infants were 6 months or younger at the beginning of their first RSV season (November to March in this region) (*1*,*2*). For the current study and based on our study objectives, we additionally excluded INSPIRE participants who were not enrolled during a well-child visit from one of the regional participating pediatric practices. The study size for INSPIRE was determined from the original power calculation for the primary outcome which required an initial sample size of 1900 participants (*3*,*4*). The current study did not have its own sample size calculation and used this existing cohort in which RSV infection was ascertained using active and passive surveillance. The Institutional Review Board of Vanderbilt University (TN, USA) granted ethical approval for INSPIRE and one parent of each participant provided written informed consent for their participation. Details of the full cohort methods have been previously published (*3*,*4*). Appendiceal Table includes the baseline characteristics of INSPIRE participants included in this analysis. Appendiceal Figure depicts the study population for this report including the INSPIRE cohort enrollment, RSV infection status, and healthcare utilization.

Ascertainment of RSV Infection

To ascertain RSV infection during infancy, we conducted passive and active surveillance during each infant's first RSV season. First, we frequently educated and reminded parents to call us at the onset of any acute respiratory symptom in the infant. Second, we contacted parents every other week by phone, by email, or in person. Lastly, we approached all infants in person who were attending one of the participating pediatric practices for an unscheduled visit (i.e., for acute illness). If an infant met prespecified criteria for an acute respiratory infection, we conducted an in-person respiratory illness assessment during which we administered a parental questionnaire, performed a physical examination, collected a nasal wash, and—in those who required an unscheduled healthcare visit (i.e., acute care outpatient, emergency department, or hospitalization)—completed a structured medical chart review to categorize illnesses as an upper or lower respiratory tract infection (URTI or LRTI, respectively). Any cases that were unclear were adjudicated by a panel of physicians blinded to the individual child. Respiratory infection severity at time of illness was measured using the Respiratory Severity Score (RSS) which is an ordinal 13-point scale based on wheezing, respiratory rate, retractions, and oxygen saturation or heart rate (*5*). The RSS ranges from 0 to 12, with higher values indicating more severe disease, and values can distinguish disease severity by LRTI versus URTI (*6*). Not all infants who met criteria for an in-person research respiratory illness visit required a healthcare visit. For example, the parents of an infant who had a fever and runny nose may not have brought their infant to a provider. However, our medical research team had set criteria to recommend an infant be seen by their provider or arrange emergency care. The nasal wash was used for the molecular detection of RSV by reverse transcription-quantitative PCR (RT-qPCR) (*7*). We also collected blood samples from all participating infants at age 1-year and measured RSV serum antibody titers by ELISA using published protocols (*8*,*9*). This technique uses RSV A and B lysate antigens produced in a human epithelial type 2 (HEp-2) cell line and detects serum antibodies against a range of RSV proteins (including the F, G, N, and P proteins). An estimated titer >200 in this assay was considered positive for RSV antibodies (*8*,*9*). When we consider the titer of antibody in cord blood from other studies, a half-life of ~30 days for maternal antibody, the age of the child at blood draw, and the antibody titer at this blood draw, it is possible that some of children the aged 9–10 months at blood draw had residual maternal antibody. We estimate that less than 10% of children might have been RSV antibody positive because of residual maternal antibody,

and do not anticipate this would significantly impact our results (*8*,*9*). Infants were then classified as infected or uninfected with RSV in the first year of life using a hierarchical categorization with mutually exclusive group membership as previously described (*4*).

Statistical Analyses

Descriptive statistics on cohort characteristic variables are presented as median (interquartile range [IQR]) or frequencies and percentages (N) as appropriate. Proportions of infection, URTI, LRTI, healthcare utilization, and hospitalizations due to RSV were calculated during infancy. We used the Wilson confidence intervals methods for the corresponding 95% confidence intervals (95% CI) (*10*). Predictors and estimated degrees of freedom were selected a priori and included the infant's sex, ever breastfeeding, daycare attendance in infancy, exposure to secondhand smoking in utero or early infancy, maternal asthma, infant birth month (referent October), presence of siblings, neighborhood percentage of people below the poverty level (percent in the census tract), insurance type (public, private, other), and the RSV study season. Infant birth month was included with restricted cubic splines to capture nonlinear association with RSV infection and contrast by month to estimate odds ratios (e.g., June versus October). The percentage of people below the poverty level by census tract was obtained from the social vulnerability index data file and linked to our cohort participants at the census tract level (*11*). We used multivariable logistic regression analyses to simultaneously include the pre-specified predictors and calculate adjusted odds ratios and 95% CI for the association of each risk factor and RSV infection status. We calculated the fraction of likelihood of the outcome (RSV infection status, infected versus not infected in infancy) explained by each risk factor based on respective partial $\chi 2$ values minus degrees of freedom from the multivariable logistic regression model. Then, we ranked factors by their relative contribution to characterize them from least to most important.

Analyses were conducted by R 4.3.1 (*12*).

Limitations

There is a potential for selection bias in our study. Among the 1,946 infants enrolled, 266 (13.6%) were excluded from the current study: 61 were excluded by design as they were not recruited during a well-child visit from a participating pediatric practice, and 205 were excluded as we could not ascertain RSV infection status including those who either triggered a respiratory

illness visit and we were not able to obtain a nasal sample for PCR, or we were unable to obtain a blood sample to measure RSV serology at age 1-year. We assessed the characteristics of mother-infant dyads by inclusion status based on RSV infection determination (infected or uninfected). The infant participants who had RSV testing performed, tended to have an older mother (median age 27 years versus 24 years, $p < 0.001$) and slightly higher birthweight (median 3425 versus 3362 g, $p = 0.021$). Those included had a slightly older gestational age distribution as a group (included 25th percentile 38.6 weeks versus not included 25th percentile 38.0 weeks, $p = 0.005$). Included infants had a higher proportion of being breastfed (77% vs 70%, $p = 0.01$) and less smoking exposure (20% versus 34%, $p < 0.001$). RSV-associated hospitalizations and RSV LRTI were identified among those in whom RSV was identified during active surveillance. While this could result in an under estimation, 1-year assessment of infant medical history would have identified LRTI or hospitalizations among infants in whom RSV infection was determined by RSV serology only. By the INSPIRE study design, not all birth months were included. Our generalizability may be limited due to our study eligibility criteria and sociodemographic characteristics that might not be generalizable to other populations (Appendiceal Table). Additionally, our cohort represents a population that may be healthier, as they are term infants, but this is a group who represents half of RSV hospitalizations and who are now for the first time eligible for RSV prevention products. There is also potential for misclassification of infants categorized as uninfected with RSV in infancy (*4*). However, such non-differential misclassification would be more likely to bias results toward a lower estimate. Both the proportion with symptomatic respiratory illness and overall RSV serologic positivity are very similar to estimates from other studies. In INSPIRE, the proportion of participants with RSV infection during infancy among those with symptoms was 30%, similar to that reported by Wildenbeest and colleagues (26%) (*13*). In addition, the proportion of infants with RSV infection in INSPIRE (53%) is very similar to a study that used twice weekly surveillance and molecular methods (56%), and identical to another study that used serologic methods to detect all RSV infections within the first year of life (53%) (*14*,*15*).

References

1. Haynes AK, Prill MM, Iwane MK, Gerber SI; Centers for Disease Control and Prevention (CDC). Respiratory syncytial virus—United States, July 2012–June 2014. MMWR Morb Mortal Wkly Rep. 2014;63:1133–6.

2. Wu P, Dupont WD, Griffin MR, Carroll KN, Mitchel EF, Gebretsadik T, et al. Evidence of a causal role of winter virus infection during infancy in early childhood asthma. Am J Respir Crit Care Med. 2008;178:1123–9. https://doi.org/10.1164/rccm.200804-579OC

3. Larkin EK, Gebretsadik T, Moore ML, Anderson LJ, Dupont WD, Chappell JD, et al.; INSPIRE Study. Objectives, design and enrollment results from the Infant Susceptibility to Pulmonary Infections and Asthma Following RSV Exposure Study (INSPIRE). BMC Pulm Med. 2015;15:45. https://doi.org/10.1186/s12890-015-0040-0

4. Rosas-Salazar C, Chirkova T, Gebretsadik T, Chappell JD, Peebles RS Jr, Dupont WD, et al. Respiratory syncytial virus infection during infancy and asthma during childhood in the USA (INSPIRE): a population-based, prospective birth cohort study. Lancet. 2023;401:1669–80. https://doi.org/10.1016/S0140-6736(23)00811-5

5. Rodriguez H, Hartert TV, Gebretsadik T, Carroll KN, Larkin EK. A simple respiratory severity score that may be used in evaluation of acute respiratory infection. BMC Res Notes. 2016;9:85. https://doi.org/10.1186/s13104-016-1899-4

6. Feldman AS, Hartert TV, Gebretsadik T, Carroll KN, Minton PA, Woodward KB, et al. Respiratory severity score separates upper versus lower respiratory tract infections and predicts measures of disease severity. Pediatr Allergy Immunol Pulmonol. 2015;28:117–20. https://doi.org/10.1089/ped.2014.0463

7. Kodani M, Yang G, Conklin LM, Travis TC, Whitney CG, Anderson LJ, et al. Application of TaqMan low-density arrays for simultaneous detection of multiple respiratory pathogens. J Clin Microbiol. 2011;49:2175–82. https://doi.org/10.1128/JCM.02270-10

8. Jadhao SJ, Anderson LJ. Detection of RSV antibodies in human plasma by enzyme immunoassays. Methods Mol Biol. 2016;1442:41–52. https://doi.org/10.1007/978-1-4939-3687-8_4

9. Jadhao SJ, Ha B, McCracken C, Gebretsadik T, Rosas-Salazar C, Chappell J, et al. Performance evaluation of antibody tests for detecting infant respiratory syncytial virus infection. J Med Virol. 2021;93:3439–45. https://doi.org/10.1002/jmv.26736

10. Wilson EB. Probable inference, the law of succession, and statistical inference. J Am Stat Assoc. 1927;22:209–12. https://doi.org/10.1080/01621459.1927.10502953

11. Centers for Disease Control and Prevention/Agency for Toxic Substances and Disease Registry/Geospatial Research, Analysis, and Services Program. CDC/ATSDR Social Vulnerability Index. 2018. https://www.atsdr.cdc.gov/placeandhealth/svi/data_documentation_download.html

12. R Core Team. (2023). R (4.3.1): a language and environment for statistical computing. R Foundation for Statistical Computing, Vienna, Austria. https://www.R-project.org

13. Wildenbeest JG, Billard MN, Zuurbier RP, Korsten K, Langedijk AC, van de Ven PM, et al.; RESCEU Investigators. The burden of respiratory syncytial virus in healthy term-born infants in Europe: a prospective birth cohort study. Lancet Respir Med. 2023;11:341–53. https://doi.org/10.1016/S2213-2600(22)00414-3

14. Munywoki PK, Koech DC, Agoti CN, Cane PA, Medley GF, Nokes DJ. Continuous invasion by respiratory viruses observed in rural households during a respiratory syncytial virus seasonal outbreak in coastal Kenya. Clin Infect Dis. 2018;67:1559–67. https://doi.org/10.1093/cid/ciy313

15. Zylbersztejn A, Pembrey L, Goldstein H, Berbers G, Schepp R, van der Klis F, et al. Respiratory syncytial virus in young children: community cohort study integrating serological surveys, questionnaire and electronic health records, Born in Bradford cohort, England, 2008 to 2013. Euro Surveill. 2021;26:2000023. https://doi.org/10.2807/1560-7917.ES.2021.26.6.2000023

Appendix Table. Baseline characteristics of INSPIRE participants in the analysis*

Characteristic	Study population, N = 1,680
Age of mother at enrollment, y	27 (23–31)
Age of child at enrollment, d	55 (16–77)
Female sex	198/1,680 (48%)
Race and ethnicity	
Black non-Hispanic	289/1,680 (17%)
White non-Hispanic	1,101/1,680 (66%)
Hispanic	152/1,680 (9%)
Other	138/1,680 (8%)
Respiratory syncytial virus season	
2012–2013	723/1,680 (43%)
2013–2014	957/1,680 (56%)
Gestational age, weeks	39 (38.6–40.0)
Birthweight, g	3425 (3,121–3,746)
Birth by cesarean section	534/1,680 (32%)
Any breastfeeding	1338/1,672 (80%)
Daycare attendance during infancy	558/1,659 (34%)
Presence of another child aged <6 y at home during infancy	834/1,680 (50%)
Exposure to secondhand smoking in utero or during early infancy	755/1,680 (45%)
Maternal asthma	319/1,679 (19%)
Type of insurance	
Federal or state	883/1,680 (53%)
Private	777/1,680 (46%)
Other	20/1,680 (1%)
Region	
Urban†	1,281/1,667 (77%)
Rural	386/1,667 (23%)

*Data presented as median (interquartile range) for continuous variables or number (%) for categorical variables.
†Includes urban and suburban.

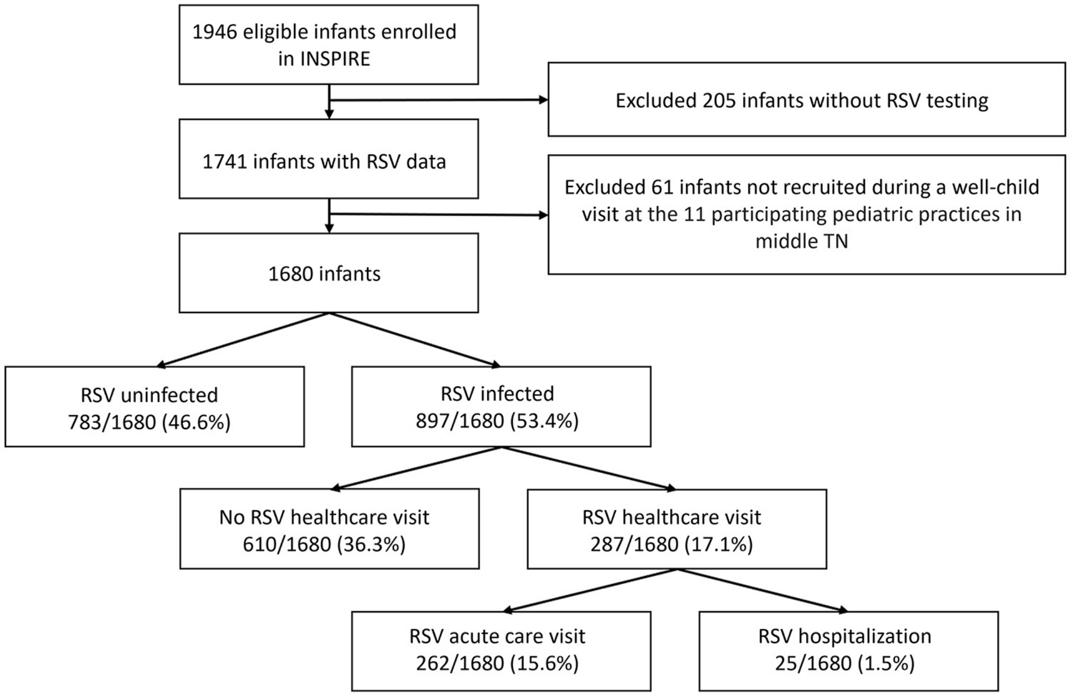

Appendix Figure. Flow diagram of enrolled and included INSPIRE cohort participants, RSV infection status, and RSV-associated healthcare utilization. The percentages represent the proportion of the 1,680 infants who met inclusion/exclusion criteria.
